# Challenges and Clinical Strategies of CAR T-Cell Therapy for Acute Lymphoblastic Leukemia: Overview and Developments

**DOI:** 10.3389/fimmu.2020.569117

**Published:** 2021-02-10

**Authors:** Xinjie Xu, Shengkang Huang, Xinyi Xiao, Qihang Sun, Xiaoqian Liang, Sifei Chen, Zijing Zhao, Zhaochang Huo, Sanfang Tu, Yuhua Li

**Affiliations:** ^1^ Department of Hematology, Zhujiang Hospital, Southern Medical University, Guangzhou, China; ^2^ The Second School of Clinical Medicine, Southern Medical University, Guangzhou, China; ^3^ State Key Laboratory of Cardiovascular Disease, Fuwai Hospital, National Center for Cardiovascular Diseases, Chinese Academy of Medical Sciences and Peking Union Medical College, Beijing, China

**Keywords:** CAR T-cell, application, challenges, access, adverse events, resistance, relapse, clinical strategies

## Abstract

Chimeric antigen receptor (CAR) T-cell therapy exhibits desirable and robust efficacy in patients with acute lymphoblastic leukemia (ALL). Stimulated by the revolutionized progress in the use of FDA-approved CD19 CAR T cells, novel agents with CAR designs and targets are being produced in pursuit of superior performance. However, on the path from bench to bedside, new challenges emerge. Accessibility is considered the initial barrier to the transformation of this patient-specific product into a commercially available product. To ensure infusion safety, profound comprehension of adverse events and proactive intervention are required. Additionally, resistance and relapse are the most critical and intractable issues in CAR T-cell therapy for ALL, thus precluding its further development. Understanding the limitations through up-to-date insights and characterizing multiple strategies will be critical to leverage CAR T-cell therapy flexibly for use in clinical situations. Herein, we provide an overview of the application of CAR T-cell therapy in ALL, emphasizing the main challenges and potential clinical strategies in an effort to promote a standardized set of treatment paradigms for ALL.

## Introduction

Chimeric antigen receptor (CAR) T-cell therapy has achieved impressive outcomes in the treatment of hematological malignancies, especially providing a potentially curative option for patients with relapsed/refractory B-cell acute lymphoblastic leukemia (R/R B-ALL). Before the emergence of CAR T-cell therapy, the prognosis of patients with R/R ALL was poor, with a 5-year survival rate of 21% in children (1) and 10% in adults (2). However, CAR T-cell therapy has improved the situation remarkably, with a high complete remission (CR) rate of 57% to 93% (**Table 1**). In 2017, the Food and Drug Administration (FDA) approved the first CAR T-cell therapy drug, tisagenlecleucel, for the treatment of R/R B-ALL patients under 25 years old (15). Afterwards, a remarkable growth spurt of clinical application was observed, and the developments of CAR T-cell therapy in R/R B-ALL are quite rapid and promising.

**Table 1:** Selected published clinical trials of CAR T-cell therapy.

Authors	Target antigen	Costimulatory domain	Number of patients	Infused cell dose per kg	Conditioning	Patients with CR(%)	Patients with relapse (%)	Patients bridging to HSCT	Patients with CRS	Patients with NT	Patients with B-cell aplasia	OS rate
Grupp et al. (3)	CD19	4-1BB	2	(0.14-1.2) × 10^7^	None/Cy+Eto	2(100%)	1(50%)	0	2(100%) Severe 50%	Unknown	2(100%)	Unknown
Maude et al. (4)	CD19	4-1BB	30	(0.76-20.6) × 10^6^	Individualized	27(90%)	7(26%)	Unknown	30(100%) Severe 27%	13(43%)	27(90%)	78% (6m)
Lee et al. (5)	CD19	CD28	20	(1 or 3) × 10^6^	Cy+Flu	14(70%)	2(14%)	10(50%)	16(80%) Severe 30%	6(30%)	None prolonged	50% (12m)
Turtle et al. (6)	CD19	4-1BB	30	(0.2 or 2 or 20) × 10^6^	Cy+ Flu/Cy/Cy+Eto	27(93%)	9(33%)	13(43%)	25(83%) Severe 23%	15(50%)	Unknown	Unknown
Gardner et al. (7)	CD19	4-1BB	43	(0.5-10) x 10^6^	Cy/Cy+Flu	40(93%)	18(45%)	11(26%)	40(93%) Severe 23%	21(49%)	Unknown	69.5% (12m)
Maude et al. (8)	CD19	4-1BB	75	(0.2-5.4) x 10^6^	Cy+Flu	61(81%)	22 (36%)	8(11%)	58(77%) Severe 47%	30(40%)	62(83%)	76% (12m)
Park et al. (9)	CD19	CD28	53	(1 or 3) x 10^6^	Cy/Cy+Flu	44(83%)	25(57%)	17(32%)	45(85%) Severe 26%	23(44%)	Unknown	50% (12.9m)
Hay et al. (10)	CD19	CD28/4-1BB	53	2 x (10^5^-10^6^)	Cy/Cy+Flu	45(85%)	22(49%)	18(34%)	40(75%) Severe 19%	12(23%)	Unknown	50% (20m/5m)^a^
Frey et al. (11)	CD19	4-1BB	35	5 × (10^7^-10^8^)	Individualized	24(69%)	Unknown	9(26%)	33(94%) Severe 18%	14(40%)	Unknown	50% (19.1m)
Fry et al. (12)	CD22	4-1BB	21	(0.3 or 10 or 30) x 10^5^	Cy+Flu	12(57%)	8(66%)	Unknown	16(76%)	6(28%)	12(57%)	Unknown
Pan et al. (13)	CD22	4-1BB	34	≤4 x 10^6^, ≤1 x 10^6^	Cy+Flu	24(71%)	5(21%)	11(32%)	31(91%) Severe 3%	6(18%)	19(56%)	Unknown
Shah et al. (14)	CD22	4-1BB	58	(0.3-3) x 10^6^	Cy+Flu	40(73%)	30(75%)	14(25%)	Unknown	Unknown	Unknown	38% (10m)

CAR, chimeric antigen receptor; CR, complete remission; HSCT, hematopoietic stem cell transplantation; CRS, cytokine release syndrome; NT, neurotoxicity; OS, overall survival; Cy, cyclophosphamide; Eto, etoposide; Flu, fludarabine; m, months; ^a^MRD-negative CR patients compared to those who did not(median OS: 20m vs 5m).

**Figure 1:**
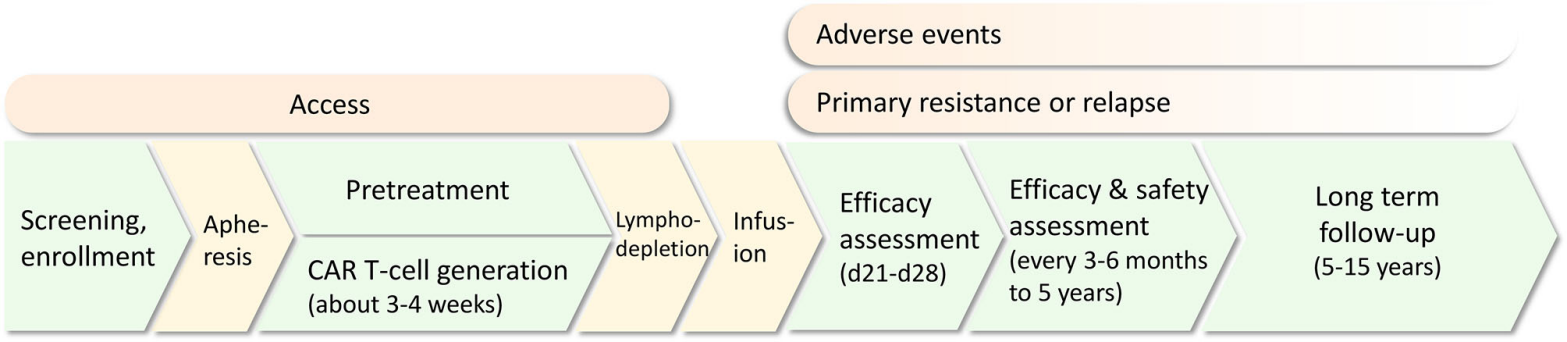
The common treatment protocol of CAR T-cell therapy for relapsed/refractory acute lymphoblastic leukemia (R/R ALL). Challenges including access, adverse events, primary resistance and relapse are present in different treatment procedures.

CAR T-cells are genetically modified T-cells that recognize specific antigens in a human leukocyte antigen (HLA)-independent method. The basic structure is composed of an extracellular domain containing single-chain variable fragments (scFvs) for antigen recognition, a transmembrane domain mainly for CAR stability support, and an endodomain including a costimulatory signaling domain (commonly CD28 or 4-1BB) and an intracellular T-cell receptor signaling domain (typically CD3ζ) for T-cell activation. Based on different intracellular domains and other modifications, CAR T-cell products can be divided into four generations. Traditional CAR T-cell administration involves patient screening, enrollment and apheresis for T-cell collection, CAR T-cell generation, pretreatment and infusion. Efficacy and safety assessments and long-term follow-up are required (**Figure 1**).

Although the procedure has been gradually standardized, there are still some limitations in several stages that impede the expanding application of CAR T-cell therapy in the clinic. Access to therapy cannot be guaranteed for every patient; adverse events highlight safety issues; and more importantly, disease relapse is an intractable issue restricting the development of CAR T-cell therapy. Correspondingly, multiple preventive and therapeutic strategies are being proposed to solve these challenges, from developing the CAR structure or optimizing clinical administration protocols. In this review, we summarize the application of CAR T-cell therapy in ALL and its development, emphasizing the main challenges and corresponding strategies. CAR engineering strategies in basic research are beyond the scope of this review; rather, we focus our attention on clinical strategies in an effort to facilitate the clinical practice of these attractive therapies.

## The Application of CAR T-Cell Therapy in B-All

Target antigen selection is the key for accurate killing. Ideal targets ought to be highly expressed in all tumor cells, with no expression in normal tissues, and do not downregulate expression under immune pressure. For ALL, at present, there are several relatively widely accepted targets in clinical applications with extraordinary outcomes; furthermore, many emerging alternative targets are being proposed and are under active research (**Table 2**).

**Table 2:** Target antigens of CAR T-cell therapy.

Antigen	CD19	CD22	CD123	CD38	CSPG4	TSLPR	CD133	CD20
Function	Helps primitive B cells differentiate into pre-B-cells and maintains the balance of mature B-cells in peripheral blood	Mediates B-cell inhibitory signals, which are essential for maintenance of the tolerance of B-cells	Helps the proliferation and differentiation of hematopoietic cells	Serves as adhesive type II transmembrane protein	Improves invasiveness and resistance of leukemia cells	Participates in the development of ALL as a tumor protein	Not detected	Promotes the cycle of B-cells
Normal tissue expression	Widely expressed in B-cell lines (from the pro-B-cell stage to plasma cells)	Widely expressed in B-cell lines (from pre-B-cell stage to mature B-cells)	Hematopoietic stem cells, dendritic cells, monocytes, endothelial cells	Lineage committed blood cells, smooth muscle cells of the lungs and liver	Not expressed in normal hematopoietic cells	Dendritic cells and CD4 + T-cells	Hematopoietic stem cells, progenitor cells	Upgrades expression rate from pro-B-cells and persists in mature B-cells
B-ALL expression	90%-100%	50%-100% in adults and 90% in children	About 80%	Partial R/R B-ALL	About 90% (MLL-r acute leukemia	5% -15% (mainly CRLF2 mutation)	MLL-r B-ALL	40% to 50% in precursor B-ALL
Progress	FDA approved	Phase 1 trials (single & dual & bispecific CARs)	Phase 1 (dual CARs with CD19)	Phase 1/2 (NCT03754764)	Preclinical (In vitro experiments)	Preclinical (xenografts murine models)	Preclinical(animal models)	Preclinical (CD19/CD20/CD22 trivalent CARs)
Application	Main CAR-T agent of CAR T-cell therapy	Alternative target and CD19/CD22 dual CARs to treat or prevent after CD19 CAR relapse	CD19/CD123 dual CARs to treat or prevent after CD19 CAR relapse	Alternative target to treat relapse after CD19 CAR cell therapy	Alternative target to treat or prevent MLL-r relapse	Alternative target for high-risk variant ALL	CD19/CD123 dual CAR to prevent relapse of MLL-r ALL	CD19/CD20/CD22 trivalent CAR to treat or prevent relapse
Reference	(16–19)	(12, 13, 20)	(21)	(22)	(23, 24)	(25–27)	(28, 29)	(22, 30, 31)

CAR, chimeric antigen receptor; B-ALL, B-cell acute lymphoblastic leukemia; MLL-r, mixed lineage leukemia rearrangement.

### CD19

CD19 is currently the most widely used and highly developed option for the treatment of B-ALL and is applied in almost 70% of CAR T-cell therapies (32, 33). CD19 is universally expressed in B-cell lines and plays an important role in the maturation of B-cells. Since the first two patients treated with tisagenlecleucel (CTL019) were reported in 2013 (3), further studies have developed rapidly. A global, multicenter, phase II clinical trial, ELIANA (NCT02435849), became a crucial study for the clinical application of CAR T-cell therapy (8), in which 75 ALL patients from 25 research centers had an overall response rate of 81% with minimal residual disease (MRD) negativity after the infusion of CTL019. The rates of event-free survival (EFS) and overall survival (OS) for 6 months were 73% and 60%, respectively, and for one year, 63% and 46%, respectively, which favorably achieved the FDA’s audit standards for approval. These data also manifested significant superiority in contrast with data of other FDA-approved agents, such as clofarabine and blinatumomab (34, 35). Simultaneously, different constructs of CD19 CAR T-cells have been tested with various outcomes (5–7, 9). A meta-analysis (36) included 35 clinical trials with different costimulatory domains, scFv clones, or T-cell origins and reported that the pooled CR was 80% (95% CI, 75.5–84.8; I²=56.96%). However, most completed trials are early phase single-arm trials. Further comparison studies are warranted to confirm the efficacy and determine associated influential factors.

### CD22

CD22 is a common alternative target in clinical trials. CD22 is also restricted to the B-cell lineage, expressed in 50% to 100% of adult ALL patients and approximately 90% of pediatric patients (37, 38). A common expression threshold of eligibility for anti-CD22-directed therapy trials is at least 20% to 30% of tumor cells (39). Pan et al. (13) reported a 70.5% (24/34) total CR rate induced by CD22 CAR T-cells on day 30. Among these patients, 91% (31/34) previously exhibited CD19 CAR T-cell therapy failure. Fry et al. (12) published initial data in 2018 that CR induced by their CD22 CAR T-cells was 57% (11/21). Recently, the study was updated to include 55 B-ALL patients (51 after prior CD19-targeted therapy) infused with CD22 CAR T-cells and adopted an improved manufacturing method. The CR rate was 70%, and the median OS was 13.4 months (95% CI, 7.7–20.3 months), validating CD22 CAR T-cell therapy as an effective salvage regimen for patients who fail CD19-targeted therapies (14). However, there are still some limitations of CD19 and CD22 CAR T-cells, which will be discussed below.

### CD123

CD123 is widely expressed in the hematological system without lineage restriction, both in normal cells such as hematopoietic stem cells, dendritic cells, monocytes, endothelial cells, and malignant cells such as acute myeloid leukemia and ALL (21). Ruella et al. (40) proved the robust potency of CD123 CAR T-cells on primitive B-ALL cells and CD19-negative B-ALL cells *in vitro* and in a murine model. Because CD123 is expressed in most CD19-negative relapsed or inherent CD19-resistant subpopulations, CD123 CAR T-cell therapy is anticipated to be an ideal prevention or remedy for post-CD19 CAR relapse. However, CD123 is also expressed on normal hematopoietic stem cells, and irreversible myeloablative impacts of CD123 CAR T-cells were reported by previous studies (41, 42). On-target off-tumor toxicity should be considered carefully when translating this therapy into clinical practice.

### CD38

CD38, an adhesive type II transmembrane protein (22), is expressed in monocytes and smooth muscle cells in the liver and lung and activates T-cells in normal tissues. It could also be detected in R/R B-ALL (43, 44) and some attempts have been made to apply anti-CD38 CAR T-cells in a phase 1/2 clinical trial (NCT03754764). Guo et al. (45) reported a preliminary case of CD38 CAR T-cells in an R/R B-ALL patient after bispecific CD19/CD22 CAR T-cell failure. CD38 CAR T-cells reduced the tumor burden in bone marrow and blood but caused uncontrollable cytokine release syndrome (CRS). Obvious off-tumor effects have been found due to CD38 expression in normal cells, especially in CAR T-cells, resulting in fratricide and short-term survival. Locking CD38 with antibodies or proteins may be capable of avoiding autolysis, ensuring the continuous proliferation and long-term potency of CD38 CAR T-cells in future clinical applications (46). Ongoing efforts to confirm persistence and safety issues of CD38 CAR T-cell therapy in the field of leukemia are underway.

### BAFF-R

B-cell activating factor receptor (BAFF-R, also known as TNFRSF13C), as the main receptor for BAFF, is responsible for B-cell maturation, survival and activation of the T-cell–mediated immune response. BAFF-R is universally expressed in mature B-cells of healthy people but abnormally expressed in precursor cells of patients with B-ALL (47–49). Turazzi et al. (50) constructed an efficient BAFF-R CAR (INVsh.BAFFR. CAR), which can proliferate and secrete cytokines to lyse ALL cell lines. Qin et al. (51) also verified the efficiency of BAFF-R CAR T-cells on CD19-negative ALL cells in blinatumomab relapse patient-derived xenografts *in vivo*. It has been suggested that BAFF-R is preserved in relapsed tumor cells since the BAFF/BAFF-R signaling pathway is essential for the survival of ALL cells and may result in a low rate of downregulated expression (52). Targeting BAFF-R or combining it with CD19 CAR is a potential direction to treat or reduce the risk of CD19-negative relapses.

### CSPG4

Chondroitin sulfate proteoglycan 4 (CSPG4, also known as neuron-glial antigen-2, NG2) is a type of single transmembrane protein that is expressed on a variety of tumors, including melanoma, breast cancer, malignant gliomas and leukemia (53–55). Regarding hematological tumors, CSPG4 is mainly expressed on KMT2A rearrangement (56–59), or the more commonly called mixed lineage leukemia rearrangement (MLL-r) phenotype. MLL-r is characterized by chromosome 11 translocation and is generally insensitive to common chemotherapy regimens with a poor prognosis (60). MLL-r ALL patients undergoing CD19 CAR T-cell therapy also have high risks of relapse due to lineage switching (6, 61). Compared with lineage-restricted targets, such as CD19 and CD22, CSPG4 can be detected in both MLL-r ALL and AML cells, and its expression is not affected by lineage switching. Harrer et al. (62) provided basic evidence for the application of CSPG4 CAR T-cells in MLL B-ALL. The constructed MLL-r leukemia cell line KOPN8 can activate cocultured CSPG4 CAR T-cells, secreting cytokines and eradicating targeted cells. More studies are warranted to further validate their therapeutic potential.

### TSLPR

Thymic stromal lymphopoietin receptor (TSLPR) is a heterodimeric complex formed by the TSLPR subunit and CD127 subunit. The former is encoded by the cytokine receptor-like factor 2 (CRLF2) gene (63). The overexpression of TSLPR in 5% to 15% of ALL patients is mainly due to CRLF2 translocations or changes in promoter regions (64–66). CRLF2 rearrangement could also cause Philadelphia chromosome-like (Ph-like) ALL, a high-risk phenotype with resistance to traditional chemotherapy and poor outcomes (64, 66). Qin et al. (25) constructed an effective anti-TSLPR CAR T-cell design to eliminate TSLPR-overexpressing ALL cells in animal models, with comparable antileukemia efficacy of CD19 CARs. It has been confirmed that abnormalities in the TSLPR signaling pathway are closely associated with cell canceration (67, 68). TSLPR is of great importance in the occurrence of leukemia cells and is less likely to downregulate expression for relapse. Therefore, TSLPR CAR T-cells are expected to become a novel therapeutic scheme for high-risk leukemia.

## Challenges

### Access

**Figure 2:**
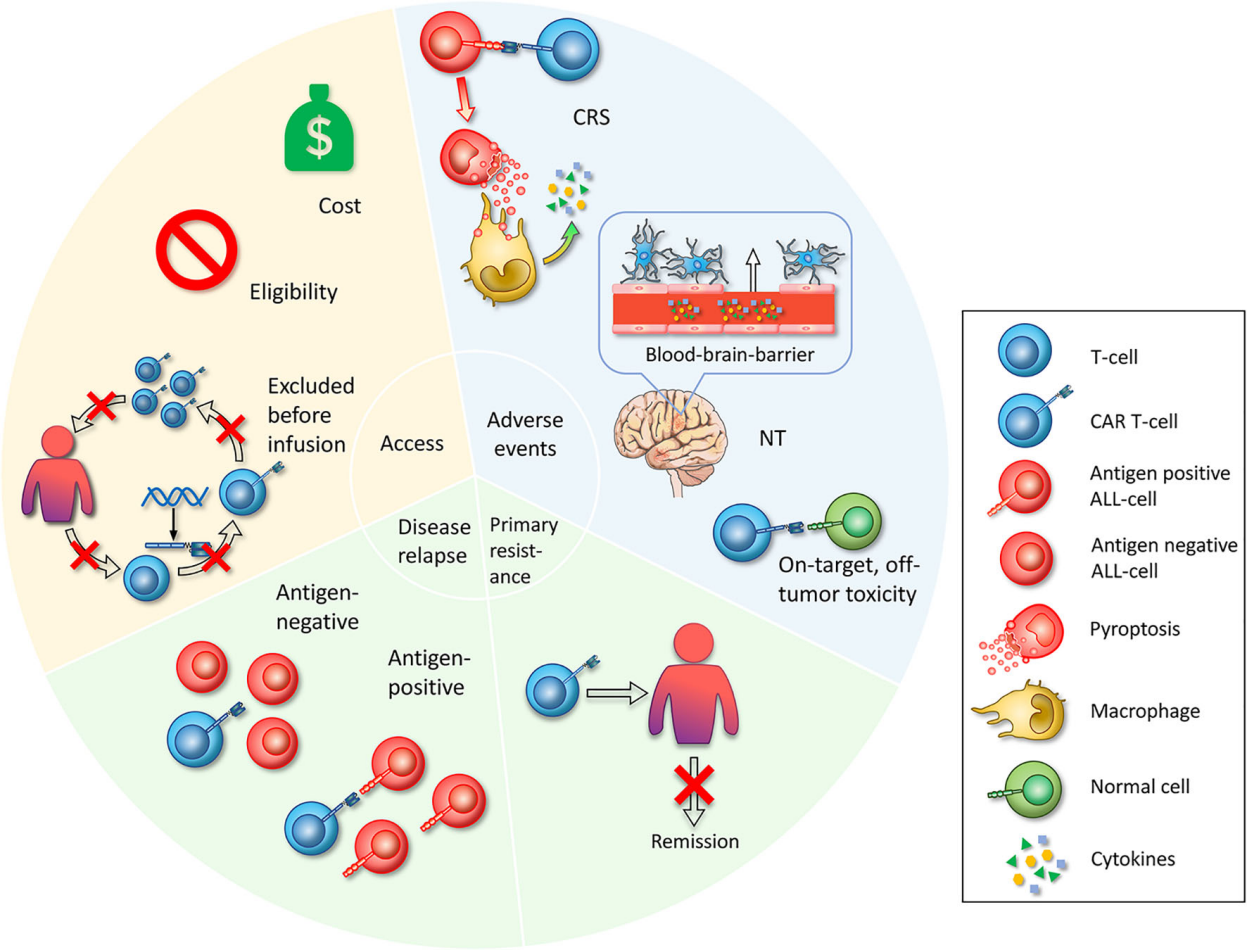
Challenges in CAR T-cell therapy. First, access is whether the patient can start CAR T-cell therapy. Limited access includes the cost of the manufacture of CAR T-cell therapy itself and other attendant expenses during the treatment is unaffordable for common patients. Eligibility, referring to specific admission or exclusion criteria set by clinical trials, impeding broad enrollment. Excluded before infusion refers to patients who have been enrolled and have experienced leukapheresis, who exhibit adverse disease progression during the preparation of CAR T-cells, or who fail to produce CAR T-cell products. The second challenge is serious adverse events that occur during the treatment with CAR T-cells. The mechanisms of CRS are that CAR T-cells specifically recognize ALL cells and lead to widespread pyroptosis, with the pyroptosis-released factors then activating macrophages to produce inflammatory cytokines. Neurotoxicity is a toxic encephalopathy state because of the disruption of the BBB endothelium. On-target, off-tumor toxicities mean that CAR T-cells recognize and attack normal cells. Third, primary resistance refers to the inability to induce remission after CAR T-cell infusion. Fourth, relapse includes antigen-positive relapse with the presence of target antigen and antigen-negative relapse with antigen loss or dim expression.

The premise of benefitting from CAR T-cell therapy is based on successful reception of effective products. Currently, the main access is through FDA-approved drugs or enrollment in clinical trials. However, several obstacles restrict patients receiving treatment, including cost issues, limitations of inclusion criteria and unexpected status of the gap period between leukapheresis and infusion (**Figure 2**).

#### Cost

CAR T-cells, as tailored and gene-editing drugs, are undoubtedly costly. Tisagenlecleucel, priced at $475,000 in the USA for a single infusion, is one of the most expensive agents for cancer treatment (69, 70). The current manufacture of CAR T-cells involves obtaining peripheral blood mononuclear cells from apheresis using anti-CD3/CD28 beads for activation, retroviral or lentiviral transduction and expansion of modified cells in bags. Expenditures mainly come from CAR T-cell generation, logistics transportation and clinical medication. In particular, GMP-graded viral vectors for CAR gene transduction place extremely high demands on professional technicians and equipment (71). Costs with medical staff and supportive care services for complication management and hospitalization should be considered simultaneously. Time cost is another aspect. The manufacturing process usually takes 12 to 17 days for the autologous cell product, during which patients are at risk of disease progression.

#### Eligibility

In addition to FDA-approved CAR T-cells, patients could also be enrolled in clinical trials. However, eligibility criteria are also strict, varying with different characters of each product. Expert consensus of CAR T-cell therapy management guidelines proposed by a multidisciplinary and interprofessional team recommended that the minimum eligibility criteria of new CAR T-cell trials should be based upon the FDA approved indications and former experience of pivotal studies (72). It has been suggested that graft-versus-host disease (GVHD), uncontrollable infections, recent donor-lymphocyte infusion (DLI) treatment, and the sites of active disease should be evaluated carefully, especially for central nervous system (CNS) pathology, due to potential neurotoxicity associated with CAR T-cell therapy (72). In addition, protocol-specific criteria included age, weight, and prior treatment status, such as the history of HSCT, the history of CD19-targeted therapy, absolute lymphocyte counts (ALCs), and liver and kidney function based on their research purposes (7, 73).

#### Excluded Before Infusion

Before infusion, patients can still be excluded from clinical trials due to infection, disease progression or production failure (8, 9). After apheresis, patients usually receive lymphodepletion, making them vulnerable to infection. ALL progresses rapidly, and as a rescue plan, enrolled patients are generally in poor condition. Therefore, they are likely to experience disease deterioration or infections during the manufacturing period. On the other hand, there are chances for manufacturing failure (8, 9). Poor T-cell quality and insufficient collection are the main barriers. T-cells collected from the peripheral blood of ALL patients are directly affected by their age, differences in antigen exposure, and chemotherapy drugs (74–76). Chemotherapy could induce T-cell deletion and/or dysfunctional metabolism through mitochondrial damage (77). Other factors include manufacturing samples that mostly contain terminal effector T-cells (T_Eff_) and effector memory cells (T_EM_) with poor proliferation capability (78), and culture systems contain suppressive components, such as myeloid-derived monocytes, inhibiting the expansion of T-cells (79). The risk of production failure caused by manufacturing technology and transportation is decreasing gradually, benefiting from the emergence of substantially successful experiences and the proposal of production guidelines (80, 81).

### Adverse Events

#### Cytokine Release Syndrome

CRS is the most common adverse event following CAR T-cell therapy, with a prevalence of 75% to 100% in patients (**Table 1**), which refers to a systemic inflammatory response mediated by the release of excessive cytokines, including IL-6, IL-1, IL-8, IFN-γ, GM-CSF, macrophage inflammatory protein-1B (MIP-1B), and monocyte chemoattractant protein-1 (MCP-1) (82, 83). The clinical manifestations can range from fever to severe complications, such as hypotension, hypoxia, capillary leakage, and multiple organ failure (84). Many parameters are associated with the incidence of CRS, including the infusion dose of CARs, tumor burden, chemotherapy regimen, and CAR construct (85–87).

IL-6, the hallmark cytokine of CRS, was greatly elevated in the serum of patients with CRS post CAR T-cell infusion. In addition, the confirmed efficacy of IL-6 and IL-6 receptor (IL-6R) antagonists for treatment underscores that IL-6 signaling instigates the amplification cytokine cascade that contributes to the pathophysiology of CRS (5, 6). IL-6 activates Janus kinases (JAKs) and the signal transducer and activator of transcription 3 (STAT3) pathway through classic cis signaling or trans signaling. The former induces pleiotropic effects on immune cells through membrane-bound IL-6R (mIL-6R), while trans signaling is activated by the soluble form of IL-6R (sIL-6R) in many cell types, including endothelial cells. This results in the production and secretion of large amounts of cytokines by downstream cells (88).

For the generation of IL-6, monocytes and macrophages are considered the main sources. Norelli et al. (82) found that depletion of circulating monocytes from mice before CD19 CAR T-cells could prevent CRS and enable the complete suppression of CRS incidence and CRS-related mortality. At the single-cell level, monocytes specifically expressed high levels of IL6 and IL-1β. On the other hand, Giavridis et al. (89) showed that macrophages are the main source of IL-6 based on RNA sequence analyses and cell enumeration data. They further hypothesized that macrophages secrete this key factor *via* engineered CD40 ligand (CD40L)-CD40 interaction between macrophages and CAR T-cells. A recent study proposed that CAR T-cell–induced pyroptosis of targeT-cells rather than apoptosis is a critical reason for CRS (90). CD19 CAR T-cells specifically recognize ALL cells and release a mass of perforin/granzyme B, thereby activating caspase 3 and further lysing highly expressed gasdermin E on ALL cells, which leads to pore-forming activity and widespread pyroptosis of ALL cells. Furthermore, pyroptotic cells release large amounts of damage-associated molecular pattern molecules (DAMPs), which activate caspase 1 for gasdermin D cleavage in macrophages, resulting in the release of proinflammatory cytokines, such as IL-6 and IL-1β and the subsequent occurrence of CRS (**Figure 2**). Ordinary tumor-specific T-cells kill B leukemic cells, leading to apoptosis, which will not activate macrophages. Another study also confirmed that CD19 CAR T-cells can cause pyroptosis of target cells through granzyme A and gasdermin B (91).

#### Neurotoxicity

Neurotoxicity (NT) is defined as a toxic encephalopathy state following CAR T-cell infusion, accompanied by confusion, unconsciousness, delirium, tremor, aphasia, seizures, and cerebral edema (92), which is another prominent toxicity occurring in 17.6% to 50% of patients (**Table 1**). NT is associated with CRS, with clinical data showing that 90% of NT is concurrent with or after CRS (4, 93).

The occurrence of NT after CD19 CAR T-cell therapy is not fully understood. The mechanism might be associated with endothelial activation and blood brain barrier (BBB) disruption (94). Some of the CRS-released cytokines, such as IFN-γ, IL-6, IL-8, and MCP-1, can activate endothelial cells (95, 96). Biomarkers of endothelial activation, such as angiopoietin-2 (ANG2), high ratios of ANG2/ANG-1, and elevated von Willebrand factor (vWF), were higher in patients with NT. Endothelial dysfunction contributes to disruption of the BBB, and the BBB was not able to shield cerebrospinal fluid (CSF) from high levels of serum cytokines, which induced stress in brain vascular pericytes and secretion of endothelium-activating cytokines and eventually resulted in severe NT (**Figure 2**) (93, 94, 97). On the other hand, a recent study revealed CD19 expression in human brain mural cells using single-cell RNA sequencing analysis and confirmed perivascular staining at the protein level. Mural cells surround the endothelium and maintain the integrity of the BBB. This finding indicated that CD19 CAR T-cells might directly attack brain mural cells and cause increased leakiness of the BBB (98). Less severe NT incidence post CD22 CAR T-cell infusion than CD19 CAR T-cells may also be associated with this finding (14).

#### On-Target, Off-Tumor Toxicities

The targets recognized by CAR T-cells are the most common tumor-associated antigens (TAAs), whereas the target antigens exist on some normal cells, which makes normal tissues inevitably attacked by CAR T-cell–specific recognition, thereby leading to on-target, off-tumor toxicities (**Figure 2**). CD19 or CD22 CAR T-cells target B-ALL cells and simultaneously damage healthy B-cells. More than 50% of patients receiving CD19 or CD22 CARs (**Table 1**) developed B-cell aplasia, plasma cell deficiency, or even severe hypogammaglobulinemia so that they were susceptible to infection due to low immunity. Myeloablation induced by CD123 CAR T-cells and fratricide killing of CD38 CAR T-cells are more thorny off-tumor toxicities than B-cell aplasia that impede their development.

#### Other Adverse Events

Other adverse events deserve attention. CAR T-cell–associated hemophagocytic lymphohistiocytosis (HLH)/macrophage activation syndrome (MAS) is attributed to a group of severe immunological disorders characterized by hyperactivation of macrophages and lymphocytes, proinflammatory cytokine production, lymphohistiocytic tissue infiltration, and immune-mediated multiorgan failure. Patients experience a wide range of clinical syndromes, including high fevers, hepatosplenomegaly, liver/renal dysfunction, coagulopathy and cytopenia. Several clinical trials have indicated that CD19 CAR T-cell therapy could induce HLH/MAS (99), and a recent CD22 CAR T-cell clinical trial also reported a 38% incidence of HLH/MAS-like toxicities at an average time of 14 days after infusion (14). Moreover, owing to the robust antitumor efficacy of CAR T-cells, massive malignant cells are dissolved and cause tumor lysis syndrome (TLS). Lytic ALL cells rapidly release large amounts of intracellular substances into the blood, which surpass the capability of liver metabolism and renal excretion, resulting in metabolite accumulation and a series of electrolyte disorders, such as hyperuricemia, hyperkalemia, hyperphosphatemia, hypocalcemia, and metabolic acidosis, which in turn even lead to life-threatening arrhythmias or acute renal failure (100).

### Primary Resistance

Primary resistance refers to the inability to induce remission after CAR T-cell infusion (**Figure 2**). It is closely related to the intrinsic T-cell functional state at the starting material (SM) stage, which is largely a consequence of their differentiation status strongly correlating with the antitumor activity of adoptively transferred T-cells (101–104). Studies have shown that at the SM stage, compared with patients who achieved partial remission (PR) or no remission (NR), patients with CR have a higher ratio of CD45RO-CD27+CD8+ T-cells and stem cell memory T-cells (T_SCM_) (105). Numerous studies have proven that generating CAR T-cells with a less differentiated phenotype, such as central memory T-cells (T_CM_) and T_SCM_, has greater *in vivo* efficacy than T_Eff_ (6, 106–108). T_SCM_ is a subset of memory T-cells with superior self-renewal capacity and can also differentiate into T_CM_ and T_EM_ *in vitro (* 109). This subtype can even facilitate CAR T-cell homing to secondary lymphoid organs and continuous proliferation (110, 111). Other phenotypes might also be associated with primary resistance. At the SM stage, LAG^high^/TNF-α^low^ CD8+ T-cells herald a high risk for primary resistance (101). Patients with CR had a lower rate of CAR T-cells with a CD8+ PD-1+ phenotype than those with PR and NR. CD8+PD-1+ CTL019 coexpresses LAG-3+ and TIM-3+, indicating poorer prognosis of patients (105).

Primary resistance may also be associated with the inherent biological characteristics of tumors. Nathan et al. (112) found that death receptor ligands in tumors, such as Fas ligand (FasL) and TNF-related apoptosis inducing ligand (TRAIL), are of critical importance for CAR T-cell cytotoxic killing. They used a CRISPR-based genome-wide loss-of-function screen in an ALL cell line under immune pressure from CD19 CAR T-cells and found that impaired death receptor signaling in ALL may lead to significantly reduced CAR T-cell cytotoxic activity and primary resistance to CAR T-cell therapy, which in turn mediate CAR T-cell dysfunction. Moreover, tumor burden serving as an indicator for primary resistance remains controversial. Some studies demonstrated that upon overexposure to target cells, CAR T-cells are more rapidly cleared and inactivated (5, 9), whereas Finney et al. found that a high antigen burden could promote the expansion of CAR T-cells (101). Moreover, some characteristics of patients, including extramedullary diseases other than CNS, increased levels of Tregs, and high-risk cytogenetic/molecular abnormalities such as E2A/PBX1, often lead to a low remission rate (113). Further studies are needed to unveil the underlying correlation.

### Relapse

Although the CR rate of ALL in CD19 or CD22 CAR T-cell treatment is as high as 57% to 93%, the relapse rate reaches 14% to 66% (**Table 1**), which becomes one of the most significant issues limiting CAR T-cell application. Relapse includes two patterns: antigen-positive relapse and antigen-negative relapse.

#### Antigen-Positive Relapse

Antigen-positive relapse (**Figure 2**) is usually associated with short persistence and low potency of CAR T-cells (4). Components of CAR constructs, such as costimulatory domains and scFv, can influence the potency and persistence of CAR T-cells. It was found in clinical studies that 4-1BB–based CAR T-cells have greater persistence than CD28-based CAR T-cells (5, 8, 114). The CD28 costimulatory domain initiated intensive cytokine release but short persistence through rapid and robust signaling of the STAT3/PI3K/AKT pathway, which can result in more differentiated memory T-cells and a reduction in mitochondrial biogenesis (115, 116). In contrast, 4-1BB–based CARs have longer persistence through tumor necrosis factor receptor-associated factors (TRAFs) and the NF-κB pathway, leading to an increase in fatty acid oxidation and T_CM_ differentiation. 4-1BB can also ameliorate the exhaustion of CAR T-cells induced by clustering of CAR scFvs and tonic CAR CD3ζ phosphorylation (117). Immunogenicity derived from murine scFv may trigger the host immune response and limit the persistence of CAR T-cells, while replacement with humanized scFv can reduce immunogenicity (118). In addition, in a study, 12 of 14 ALL patients achieved CR treated with low-affinity scFv CAR T-cells, which showed better proliferation than higher-affinity scFv (119).

The choice of T-cell subset at the SM stage will also influence antitumor efficacy and persistence (101, 105). CD8+ CAR T-cells exhibited higher lytic activity than CD4+ CAR T-cells, while the production of abundant IL-2 by CD4+ CAR T-cells might augment the proliferation and efficacy of CD8^+^ CAR-T-cells. A defined 1:1 CD4/CD8 ratio is the most widely used, and the optimal CD4/CD8 ratio is under intensive investigation (120). In addition, age-related T-cell changes reflected by gene expression patterns and secretory profiles might influence CAR T-cell persistence (121). A study showed that CAR T-cells derived from young donors had greater expansion ability with more memory-like phenotypes but inferior cytotoxicity than those derived from geriatric donors (122). Moreover, c-Jun overexpression (123) and the immune-suppressive bone marrow microenvironment (124) are likely to be parameters related to antigen-positive relapse in CAR T-cell therapy.

#### Antigen-Negative Relapse

Antigen modulation or loss on the surface of tumor cells, which makes them incapable of being recognized by CAR T-cells, is another pattern of relapse (**Figure 2**). The mechanism by which 10% to 20% (125) of patients develop antigen-negative relapse following CD19 CAR T-cell treatment is complicated, including gene mutation, selective splicing, lineage switching, immune selection, trogocytosis and antigen masking. Sotillo et al. (126) found that tumor cells in relapsed patients can induce epitope loss through frameshift mutations and selective splicing. Although CD19 of this subtype can also transmit signals, it can no longer be recognized by FMC63 on CD19 CAR T-cells due to the lack of exon 2 expression. SRSF3 is one of the splicing factors whose function is to retain exon 2. Insufficient expression of SRSF3 in relapsed ALL cells may be a major reason for skipping the exon 2 isoform of CD19 (126). Lineage switching is another mechanism related to antigen loss. Some patients develop myeloid leukemia relapse, with an altered antigen expression profile. Generally, ALL patients with KMT2A (MLL) rearrangement develop myeloid conversion under CD19 immune pressure (6, 61). BCR-ABL1-positive and TCF3-ZNF384 fusion-positive B-ALL patients have also been found to undergo myeloid transformation after CD19-targeted immunotherapy (127, 128).

Immune selection means that under the pressure of CD19 CAR T-cells, inherently resistant CD19-negative leukemia cells are retained and evolve into a dominant clonal community. Fischer et al. (129) revealed that the total deletion (ex2-isoform) and partial deletion of exon 2 (ex2part-isoform) of CD19 in B cells, which cannot be recognized by CD19 CAR T-cells, existed in CD19+ B-ALL patients and healthy people before treatment. Grupp et al. (3) performed a flow cytometry test on a CD19-negative relapse case after CAR T-cell therapy and validated that CD19-negative clones pre-existed. Furthermore, in B-ALL xenograft studies, Hamieh et al. (130) reported trogocytosis phenomena in which CD19 antigen is transferred from ALL cells to CAR T-cells, thus resulting in antigen escape of ALL cells as well as fratricide killing of CAR T-cells. Intriguingly, there is a clinical case of mask CD19 epitope relapse (131), which means that the CAR gene was accidentally introduced into B leukemia cells, masking the CD19 epitope from detection of flow cytometry and recognition of CD19 CAR T-cells.

Regarding CD22 CAR T-cell therapy, there are also cases of CD22-negative or CD22-dim relapse. Because the potency of CD22 CAR T-cells is greatly affected by cell surface antigen density, ALL cells below a certain threshold can escape. In the trial of Fry et al. (12), seven of eight relapsed patients had a decline in CD22 site density with no CD22 gene mutation or mRNA change observed, indicating that the downregulation mechanism may be related to posttranscriptional regulation.

## Clinical Strategies

### For Access

To overcome the limitations of tailored products, numerous optimization strategies for manufacturing techniques have been proposed. Using an automation system (CliniMACS Prodigy System) (132), nonviral transposon-based systems (133–135), a type II CRISPR/Cas9 system (136) and transferring CAR coding sequences *in vivo (* 137, 138) could reduce the manufacturing time and cost as well as manifest the high potency and proliferation capacity of CAR T-cells. A new platform, “FasT”, using electroporation to transduce the CAR gene and shortening manufacturing time to 24 h, generated less differentiated phenotypes with superior expansion capacity in a first-in-human clinical trial (139). Moreover, recent advances have paid intensive attention to allogeneic, off-the-shelf CAR T-cells.

#### Allogeneic, Off-the-Shelf CAR T-Cell

To solve the problems of autologous T-cells with poor quantity and quality, allogeneic healthy donor-derived T-cells have become an attractive alternative. In Zhang’s study (140), six R/R B-ALL patients who received donor-derived CAR T-cells stayed alive with complete donor chimerism and remained in MRD-negative remission with a median follow-up of 243.5 days. Tu et al. (141) enrolled three R/R ALL patients in the study, and all reached CR after administering pooled donor-derived fourth generation CAR T-cells targeting CD19 and CD123. A meta-analysis showed that the pooled CR rate of different allogeneic CD19 CAR T-cell trials was 55% (95% CI, 30.6–79.0%), the pooled CRS rate was 53.9% (95% CI, 10.7–94.2%), and the pooled NT rate was 3.1% (95% CI, 0.0–23.0) (36).

**Figure 3:**
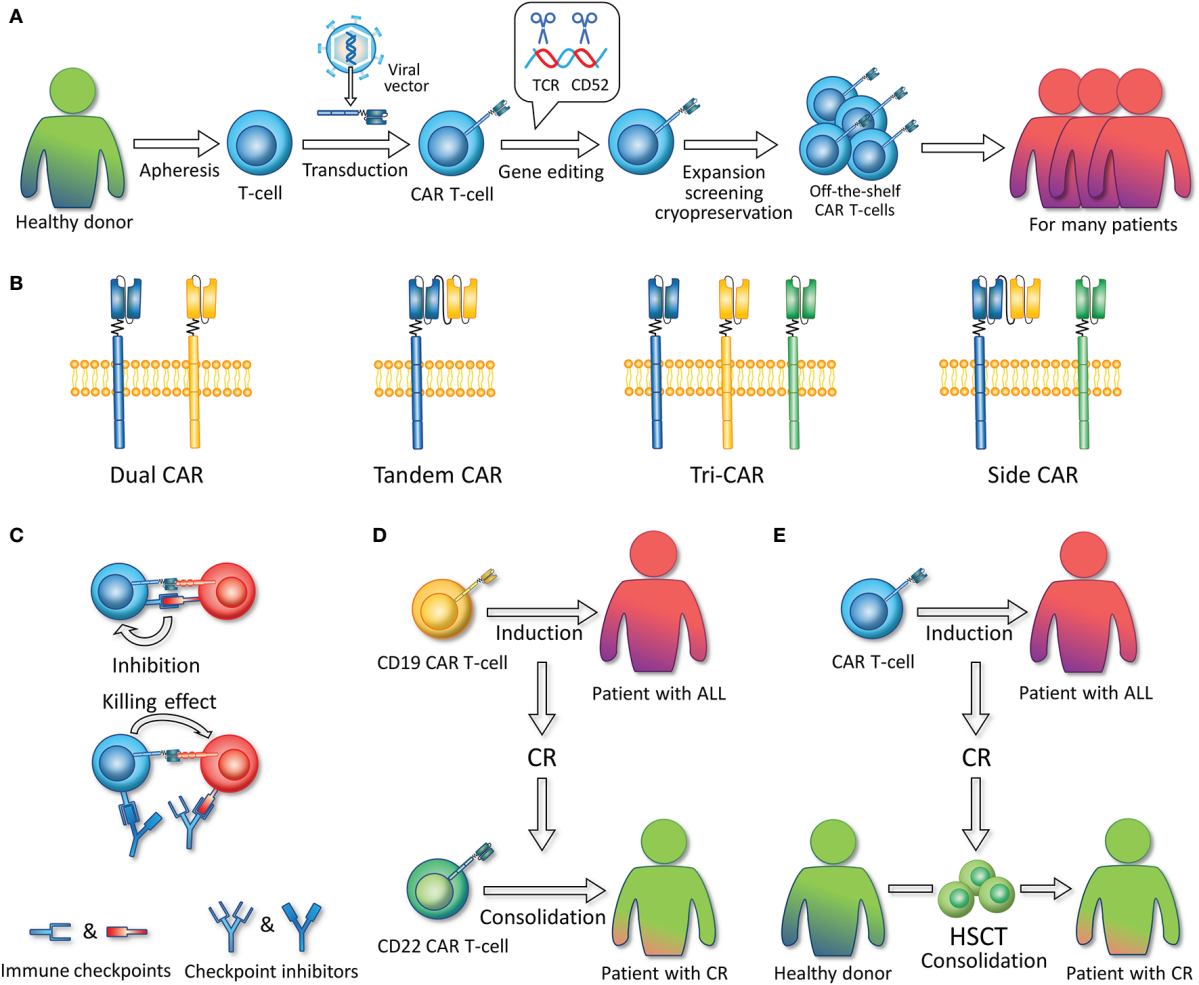
**(A)** Allogeneic, off-the-shelf CAR T-cells. The figure illustrates the general preparation process of allogeneic, off-the-shelf CAR T-cells. T-cells are extracted from healthy donors. Viral vectors are used to introduce CAR-encoding genes into T-cells. Gene editing technology is used to remove the gene fragments encoding TCR and CD52. CAR T-cells are expanded, screened, and cryopreserved. These CAR T-cell products can serve as timely treatment for many patients. **(B)** Multi-antigen targeted CAR. Dual CAR refers to two different mono-CARs in one T-cell. Tandem CAR refers to a CAR structure that contains two single-chain variable fragments. Tri-CAR T-cell coexpresses three different mono-CARs on a single T-cell. Side CAR T-cell expresses a Tandem CAR and a mono-CAR. **(C)** In combination with immune checkpoint inhibitors. Immune checkpoint inhibitors such as PD-1/PD-L1 inhibitors can specifically block the binding of CAR T-cell or ALL-expressed immune checkpoint molecules to the corresponding receptors on CAR T-cells, thereby enabling CAR T-cell activation and the killing of tumor cells. **(D)** Sequential infusion. CD19 CAR T-cells are first infused to induce CR, and after patients achieve CR, CD22 CAR T-cells are infused as consolidation. **(E)** Bridging to HSCT. CAR T-cells are infused to induce CR, and after patients achieve CR, hematopoietic stem cell transplantation (HSCT) is conducted as consolidation.

Recent advances in allogeneic CAR T-cells have focused on off-the-shelf products, also called universal CAR (UCAR). The third-party products are based on a detachable, universal, programmable CAR system and gene editing technology (142), making standardized batch preparation possible (**Figure 3A**). In a process superior to case-by-case manufacture, recipients with rapidly progressive diseases could have access to UCAR T-cells in time at a lower cost, which would make the UCAR more competitive in the future market. Ongoing UCAR19 clinical trials, PALL (NCT02808442) and CALM (NCT02746952) (143), have reported that 14 (66.7%) of the 21 R/R B-ALL patients who received UCART19 infusion achieved CR/CRi. The phase 1 trial of UCAR22 is also underway (NCT04150497). Hu et al. reported the latest clinical results of a CRISPR/Cas9-engineered universal CD19/CD22-targeting CAR T-cell product (CTA101) in B-ALL patients. A total of 5/6 (83.3%) patients achieved MRD-CR with manageable adverse events (144).

Nonetheless, the application of allogeneic CAR T-cells met additional challenges, including GVHD and graft rejection mediated by the presence of class I HLA (HLA I) and HLA II overexpression (145). Gene editing technology (142) and protein expression blockers (PEBLs) (146) are expected to be used for T-cell receptor (TCR) modulation to prevent GVHD and to date, severe GVHDs have rarely been reported in clinical trials (147, 148). In terms of graft rejection, the sources of third-party T-cells could be selected seriously from donors with high compatibility of HLA (i.e., from previous allo-HSCT donors). Simultaneously, chemotherapy regimens such as Cy/Flu for lymphodepletion and serum treatments such as the anti-CD52 monoclonal antibody alemtuzumab are advocated to suppress immune cells, which can result in long-term lymphodepletion and thereby reduce allo-rejection (149), while UCAR can be preserved for the knock-off of the CD52 gene. However, the optimal intensity and duration of immunosuppression remain to be determined and are required careful evaluation in clinical trials.

### For Adverse Events

#### For Cytokine Release Syndrome

Recent consensus on grading and management for CRS was proposed (72, 150). Finding potential biomarkers to predict CRS is also of importance. Studies suggest that IFN-γ, IL-6, and sIL2Rα are strongly correlated with severe CRS (33). The cytokine combination of sgp130, MCP1 and eosinophil chemokines in predicting severe CRS has a sensitivity of 86% and a specificity of 97% (33). For prevention, CRS can be controlled more effectively with a fractionated dosing scheme of CAR T-cells. In a trial by Frey et al. (11), patients in the high-dose fractionated group received infusion with 10% of the dose on day 1 (D1), 30% on D2, and 60% on D3. D2 and D3 were withheld for early signs of CRS, such as fever. Compared with the high-dose single infusion and low-dose group, the high-dose fractionated group had a superior CR rate (50% vs. 33% vs. 90%, P=0.004) and lower CRS incidence (50% vs. 22% vs. 5%, P=0.017).

With regard to management, the IL-6 receptor blocker tocilizumab, which has been approved by the FDA for the treatment of CRS (151), is the first-line medication for CRS without affecting the efficacy of CAR T-cells (152). Other drugs, such as siltuximab (an IL-6 blocker) (153), anakinra (an IL-1 receptor blocker) (154), and dasatinib (a tyrosine kinase inhibitor) (155), have been applied in clinical trials with significant effectiveness. For the administration of corticosteroids, their impact on CAR T-cell activity remains controversial (156, 157). When corticosteroids are used, the dose should be controlled and individualized according to the patient’s response.

#### For Neurotoxicity

Recent grading and management guidelines for NT were developed (72, 150). For clinical parameters, Ang-2 and vWF can estimate the occurrence of NT. Low platelet counts (PLT<60) are significantly associated with severe NT (158). MCP-1 proved to be the best predictor of severe NT within 36 h after CAR T-cell administration, with high specificity and sensitivity (94). With regard to the treatment of NT, tocilizumab has limited efficacy due to its difficulty in penetrating the BBB and even increasing the CSF IL-6 level because of the peripheral blockade of IL-6R (94, 159). For this reason, some agencies regard corticosteroids, such as dexamethasone, as the first-line therapy for NT (160), but the thresholds for administration and dosing schemes vary and have not been prospectively compared. On the other hand, CAR engineering strategies to overcome CRS and NT have been proposed, including “on/off” switch systems, suicide gene systems and direct antagonism approaches (161, 162).

#### For On-Target, Off-Tumor Toxicities

B-cell aplasia driven by CD19 or CD22 CAR T-cell therapy is tolerated and treatable. The administration of antibiotics and gamma globulin is capable of preventing infections and improving immunity. Furthermore, repeating vaccine series are a promising direction for patients who have achieved B-cell recovery, and this remains an active area in upcoming research (163). Myeloablation caused by CD123 CAR T- cells is risky and requires high caution, and treatment must be terminated if necessary. Generally, selecting safer TAAs and optimizing CAR constructs, such as improving specificity through targeting multiple antigens, logic gating and conditional expression systems, are the ultimate strategies to avoid on-target, off-tumor toxicities (161, 162, 164).

#### For Other Adverse Events

At present, diagnostic criteria for CAR T-cell–related HLH have been proposed (99). Allopurinol can be used for prevention before cell infusion, and IL-6 inhibitors and glucocorticoids can serve as effective medications (163). Anakinra and corticosteroids were used in a trial, and all treated participants had alleviation of HLH/MAS-like toxicities without any apparent negative impact on response or CAR T-cell expansion. In addition, etoposide and cytarabine may have therapeutic effects on HLH (165–167). For TLS, in addition to applying high-dose corticosteroids or corresponding cytokine inhibitors such as tocilizumab for treatment, reducing the infusion dose or administering different types of CAR T-cell populations can alleviate the clinical symptoms (100).

### For Primary Resistance and Relapse

Engineering strategies to improve the persistence and antitumor activity of CAR T-cells have been reviewed recently (161, 162), which is not the focus of our article.

#### Multiantigen-Targeted CAR T-Cell

Multiantigen-targeted CAR T-cells are a promising direction to overcome single antigen relapse issues since the “OR” Boolean logic gate is utilized to activate T-cells in the presence of either validated antigen. It mainly refers to dual CAR T-cells, bispecific CAR and trivalent CAR T-cells (**Figure 3B**). Dual CAR T-cells are designed to contain two different CARs on one T cell. CD19/CD22 and CD19/CD123 dual CAR T-cells for ALL have been successfully constructed and show greater potency than single CAR T-cells or a pool of both CAR T-cells in mice (40, 130, 168). Amrolia et al. (169) infused CD19/CD22 dual CAR T-cells to treat seven R/R ALL children, with a 100% molecular remission rate and a favorable safety profile. No more than grade 3 CRS or grade 2 neurotoxicity was reported.

Bispecific CAR refers to a CAR protein containing two scFv domains, which is also called Tandem CAR (TanCAR). The CD19/CD20 bispecific CAR T-cell constructed by Martyniszyn et al. was effective on a mixed CD19+CD20+/CD20-negative phenotype from the blood and bone marrow of transplanted mice, while anti-CD20 CAR T-cells left CD20-negative leukemic cells behind without curing the disease (170). In the trial of Dai et al. (171), six out of six patients with R/R B-ALL achieved MRD-negative CR after infusion of CD19/CD22 bispecific CAR T-cells, with one patient with mild CRS and no patient with NT. Since CD133 is expressed in MLL-r B-ALL cells, preclinical studies have also proposed the application of CD19/CD133 bispecific CAR T-cells (29), with the findings showing the synergistic effect of CD19 and CD133 and that T-cells simultaneously targeting CD19 and CD133 possess greater recognition efficacy than single-targeted T-cells *in vitro* and mouse models.

There are two patterns of trivalent CAR T-cells: TriCAR expressing CD19 CAR, CD20 CAR, and CD22 CAR on a single T-cell and side CAR expressing CD20/CD19 TanCAR combined with CD19 CAR (31). Fousek et al. (172) constructed CD19/CD20/CD22 TriCAR T-cells that manifested strong killing activity on both ALL cells and CD19-negative relapsed cells *in vitro* and in murine models, while CD19 CAR T-cells were ineffective.

Nonetheless, there are CD19-negative/CD22-low relapse cases after both CD19/CD22 dual and bispecific CAR T-cell–induced CR (169, 171), in which the mechanism remains to be elucidated. In addition, poor T-cell persistence is also a relatively common situation and contributes to a proportion of relapses. It is worth noting that membrane binding location, linker length between the heavy and light chains and other spatial considerations are all important for the efficacy and safety of CAR T-cells, which is more complex in the incorporation of multiple scFvs (51, 173, 174). More basic studies are required to optimize the structure of multitargeting CARs in pursuit of better clinical outcomes. On the other hand, the utilization of the “AND” “NOT” logic-gated system in multi-CAR could serve as a promising strategy for avoiding attacks on normal tissue and alleviating on-target, off-tumor toxicities (175).

#### In Combination With Immune Checkpoint Inhibitors

Immune checkpoints (PD-1, CTLA-4, TIM-3, etc.) serve as a brake on immune cell overactivity and prevent autoimmune reactivity, whereas tumor cells can escape human immune surveillance by upregulating the expression of immune checkpoints, thereby leading to tumor recurrence (176–178). On this basis, immune checkpoint inhibitors can be used to regulate the intrinsic T-cell functional state and promote the efficacy of CAR T-cells. Among them, inhibitors (e.g., nivolumab, pembrolizumab or atezolizumab) targeting the programmed cell death 1 receptor (PD-1) and its ligand PD-L1 have attracted attention (**Figure 3C**), and clinical trials in combination with CAR T-cell therapy are underway. In the study by Maude et al. (179), four children with relapsed B-ALL after CD19 CAR T-cell treatment presented poor CAR T-cell persistence. Subsequently, they received the PD-1 inhibitor pembrolizumab (PEM), and increased persistence of CAR T-cells in circulation was detected by flow cytometry. Li et al. (180) reported 13 pediatric patients with relapsed B-ALL who received CD19 CAR T-cells and PEM in combination. Enhanced CAR T-cell function was observed in three of the patients, and two partial and two complete responses were observed in four patients who previously had relapse or no response for CAR T-cells after the administration of PEM. Remarkably, significant CAR T-cell proliferation was detected in one patient within days of pembrolizumab administration. However, thus far, few clinical trials have evaluated the efficacy of CAR T-cells and PD-1 blockers in combination, and the rationality of this combination needs to be further clarified.

#### Sequential Infusion

Sequential infusion refers to the process of infusing CD19 CAR T-cells as induction to CR, followed by infusion of CD22 CAR T-cells for consolidation (**Figure 3D**). Compared with dual CAR T-cells and bispecific CAR T-cells, this two-cycle therapy is more convenient and cost-effective because it does not require the generation of complex CAR T-cell products (181). Wang et al. (182) conducted a pilot study on 51 ALL patients to evaluate the effectiveness and safety of the sequential infusion of two monospecific CAR T-cells, CD19 and CD22. Drawing on the findings that the MRD-negative rate was 96.0%, the median progression-free survival (PFS) was 13.6 months, and the median OS was 31 months with no relapse of antigen loss observed. In addition, Pan et al. (183) performed sequential infusion on 20 pediatric patients with R/R B-ALL, and the median interval between the two cycles of infusion was 1.65 months. During the 20-month follow-up period, 17 patients remained in CR, and three patients relapsed, resulting in a leukemia-free survival (LFS) rate of 79.5% at 12 months and OS rates of 92.3% at 18 months. In addition, regarding adverse events, 4 of 20 patients developed no CRS; mild or moderate (grade 1–2) CRS was observed in 15 of 20 patients, and grade 1 NT occurred in 3 of 20 patients, indicating that sequential infusion of two CAR T-cell products is a safe strategy. The data from the above clinical trials show that sequential infusion of CD19 and CD22 CAR T-cells is effective and safe and may be a feasible strategy to prevent relapse of single-targeted CAR T-cell therapy. However, the limited CAR T-cell persistence and the interval lymphodepleting chemotherapy that probably eradicates previous CAR T-cells should be considered, and longer follow-up is needed (181).

#### Consolidative HSCT

HSCT is considered to be the only potentially curative therapy for R/R B-ALL (184, 185). Serving it as a consolidative therapy after CAR T-cell–induced CR may improve the long-term outcome (**Figure 3E**). However, whether HSCT after CAR T-cell therapy benefits patients remains controversial. Park et al. (9) reported that 17 of 53 patients received allo-HSCT after 19–28z CAR T-cell therapy, and their OS or LFS was not significantly different from those who did not undergo HSCT. In contrast, there is accumulating evidence supporting the benefit of consolidative HSCT (186–188). A multicenter retrospective study indicated that haploidentical HSCT after CD19 CAR T-cell therapy could benefit patients, with great improvement in LFS and OS compared with the nontransplant group (65.6% versus 32.8% P < 0.001; 77.0% versus 36.4%, P < 0.001) and no increased risk of treatment-related toxicity compared with previous values. It also identified pretransplant MRD negativity as an important independent predictor of high LFS and OS (186). Gu et al. (187) reported similar conclusions after analyzing 56 adults with R/R Ph+ ALL who received allo-HSCT post-CD19 CAR T-cell therapy. Moreover, they further compared the 2-year treatment-related mortality between the transplant and nontransplant groups and detected no significant difference (14.3%, CI 7.6–21% vs. 9.8%, CI 3.2–16.4%, p =0.804), confirming the safety of this therapy. Bridging CD22 CAR T-cells to HSCT also seems to be feasible; 11 of 24 CR patients received transplantation after CD22 CAR T-cell therapy, with a one-year LFS rate of 71.6% (95% CI, 44.2–99.0) and a 1-year relapse rate of 9.1% (95% CI, 0–26.2), while four of the seven CR patients without further treatment had a relapse at 1.7 to 6 months (13). More randomized controlled studies with longer follow-ups are warranted to verify the efficacy and safety of these strategies.

## Discussion

In this article, we reviewed the whole process of CAR T-cell administration in ALL patients, with a focus on its clinical application, existing challenges and clinical coping strategies. Regardless of the verified effectiveness, CAR T-cell therapy still faces certain challenges. Resistance and relapse are the main barriers restricting the development of CAR T-cell therapy. The underlying mechanism has not been unveiled completely, especially for antigen-negative relapse. More studies are warranted to clarify them to promote the proposal of potential strategies. On the one hand, CAR engineering strategies are a critical part of tackling antigen-positive relapse issues by enhancing the potency and persistence of T-cells so far. On the other hand, clinically, multiantigen-targeted CAR T-cells, combinatorial therapy and sequential therapy are in the spotlight. Multiantigen-targeted CAR T-cells require the search for more rational target combinations and optimization of multi-CAR constructs to augment the specific killing effects without impairing the efficacy. In terms of combinatorial therapy, combining checkpoint inhibitors, antitumor vaccines or other novel agents may exert synergic effects, but further clinical trials are required to confirm their efficacy and safety. In sequential therapy, whether to bridge HSCT is a hotspot. An increasing number of studies have recently demonstrated its benefits, especially for patients reaching MRD-negative CR after CAR T-cell therapy. Further confirmation of the specific indications, such as age, high-risk mutation, disease risk stratification, CAR T-cell persistence *in vivo*, B-cell aplasia duration and donor type of transplantation, is necessary.

With regard to safety issues, CRS and NT deserve intensive attention, and their mechanisms are not completely understood. A growing number of *in vitro* and animal experiments have been launched to better characterize these systemic cytokine toxicities. It has been confirmed that the cytokine storm is the result of multicell crossover, while the interactions between CAR T-cells, targeted cells, other immune cells, and various cytokines are worthy of further study. Even though consensus on grading and management is introduced, there still exists controversy in individualized medication. Novel drugs and optimization schemes are needed. Additionally, off-the-shelf CAR T-cells may be a promising direction to mitigate the obstacle of access and promote the commercialization process, while manufacturing techniques and the efficacy of the products need further improvements. Generally, the challenges brought by CAR T-cell therapy in turn promote its development. In the future, more innovative and clinical controlled studies are needed to optimize and confirm this promising therapy.

## Author Contributions

XXu, ST and YL designed the study. XXu, SH, XXiao, and QS drafted the manuscript. QS, XXiao, XL, and SH prepared the tables and figures. SC, ZZ, and ZH participated in data collection. All authors participated in the revision of the manuscript. All authors contributed to the article and approved the submitted version.

## Funding

This research was supported by grants from the Clinical Research Startup Program of Southern Medical University by High-level University Construction Funding of Guangdong Provincial Department of Education (no. LC2016ZD027), the Guangdong Science and Technology Department (no. 2017A020215183), the Major Program for Health Medical Collaborative Innovation of Guangzhou (no. 201704020216), research funds from Natural Science Foundation of Guangdong Province, China (no. 2018B030311042) and Frontier Research Program of Guangzhou Regenerative Medicine and Health Guangdong Laboratory (no. 2018GZR110105014).

## Conflict of Interest

The authors declare that the research was conducted in the absence of any commercial or financial relationships that could be construed as a potential conflict of interest.
